# *Ziziphus jujuba* Miller Ethanol Extract Restores Disrupted Intestinal Barrier Function via Tight Junction Recovery and Reduces Inflammation

**DOI:** 10.3390/antiox13050575

**Published:** 2024-05-07

**Authors:** Ye Jin Yang, Min Jung Kim, Ho Jeong Lee, Won-Yung Lee, Ju-Hye Yang, Hun Hwan Kim, Min Sup Shim, Ji Woong Heo, Jae Dong Son, Woo H. Kim, Gon Sup Kim, Hu-Jang Lee, Young-Woo Kim, Kwang Youn Kim, Kwang Il Park

**Affiliations:** 1 Departments of Veterinary Medicine, Gyeongsang National University, Jinju 52828, Republic of Korea; yang93810@gnu.ac.kr (Y.J.Y.); minjung0102@gnu.ac.kr (M.J.K.); hunskim@gnu.ac.kr (H.H.K.); hujiw7806@gnu.ac.kr (J.W.H.); beeast0070@gmail.com (J.D.S.); woohyun.kim@gnu.ac.kr (W.H.K.); gonskim@gnu.ac.kr (G.S.K.); hujang@gnu.ac.kr (H.-J.L.); 2 Gyeongnam Bio-Health Research Support Center, Gyeongnam Branch Institute, Korea Institute of Toxicology (KIT), 17 Jeigok-gil, Jinju 52834, Republic of Korea; hojeong.lee@kitox.re.kr; 3 School of Korean Medicine, Wonkwang University, Iksan 54538, Republic of Korea; wonyung21@wku.ac.kr; 4 Korean Medicine (KM)-Application Center, Korea Institute of Oriental Medicine, 70 Cheomdanro, Dong-gu, Daegu 41062, Republic of Korea; jjuhye@kiom.re.kr; 5 Department of Biochemistry and Molecular Genetics, College of Graduate Studies, Midwestern University, 19555 N. 59th Ave., Glendale, AZ 85308, USA; mshim@midwestern.edu; 6 School of Korean Medicine, Dongguk University, Gyeongju 38066, Republic of Korea

**Keywords:** *Ziziphus jujuba* Miller, anti-inflammation, intestinal barrier function, tight junction, inflammatory bowel disease

## Abstract

Inflammatory bowel disease (IBD) is a chronic inflammatory condition caused by the disruption of the intestinal barrier. The intestinal barrier is maintained by tight junctions (TJs), which sustain intestinal homeostasis and prevent pathogens from entering the microbiome and mucosal tissues. *Ziziphus jujuba* Miller (*Z. jujuba*) is a natural substance that has been used in traditional medicine as a therapy for a variety of diseases. However, in IBD, the efficacy of *Z. jujuba* is unknown. Therefore, we evaluated ZJB in Caco2 cells and a dextran sodium sulfate (DSS)-induced mouse model to demonstrate its efficacy in IBD. *Z. jujuba* extracts were prepared using 70% ethanol and were named ZJB. ZJB was found to be non-cytotoxic and to have excellent antioxidant effects. We confirmed its anti-inflammatory properties via the down-regulation of inflammatory factors, including inducible nitric oxide synthase (iNOS) and cyclooxygenase-2 (COX-2). To evaluate the effects of ZJB on intestinal barrier function and TJ improvement, the trans-epithelial electrical resistance (TEER) and fluorescein isothiocyanate-dextran 4 kDa (FITC-Dextran 4) permeability were assessed. The TEER value increased by 61.389% and permeability decreased by 27.348% in the 200 μg/mL ZJB group compared with the 50 ng/mL IL-6 group after 24 h. Additionally, ZJB alleviated body weight loss, reduced the disease activity index (DAI) score, and induced colon shortening in 5% DSS-induced mice; inflammatory cytokines, tumor necrosis factor (TNF)-α, and interleukin (IL)-6 were down-regulated in the serum. TJ proteins, such as Zonula occludens (ZO)-1 and occludin, were up-regulated by ZJB in an impaired Caco2 mouse model. Additionally, according to the liquid chromatography results, in tandem with mass spectrometry (LC-MS/MS) analysis, seven active ingredients were detected in ZJB. In conclusion, ZJB down-regulated inflammatory factors, protected intestinal barrier function, and increased TJ proteins. It is thus a safe, natural substance with the potential to be used as a therapeutic agent in IBD treatment.

## 1. Introduction

Inflammatory bowel disease (IBD) has a high rate of prevalence in both the Western world and East Asia [1]. IBD degrades the health of patients, leading to massive economic and social burdens. It has a significant impact on the physical, psychological, and social aspects of life, and depression and anxiety are usually increased in these patients [2]. Thus, it is important to treat and manage IBD to ensure a good quality of life for the patient [3]. Nevertheless, concerns remain regarding the etiology of ulcerative colitis (UC), the effectiveness of current treatments, and the disease’s relevance to and influence on patients’ health and social life.

IBDs, including Crohn’s disease (CD) and UC, are chronic, disabling gastrointestinal (GI) disorders [4]. The two main forms of IBD involve an interaction between environmental factors in the intestinal lumen and inappropriate host immune responses in genetically predisposed individuals. UC involves relapsing and remitting mucosal inflammation, which frequently starts in the rectum and progresses to the colon’s proximal segments. However, CD constitutes a chronic, relapsing, systemic inflammatory disease that mainly affects the gastrointestinal tract, along with extra-intestinal manifestations and associated immune disorders [5]. Both UC and CD are caused by defects in the intestinal epithelial barrier function—a characteristic feature of IBD. The intestinal mucosal barrier strikes a balance between absorbing vital nutrients and blocking the entry of hazardous substances from the luminal environment, such as pathogens, toxins, and allergens, and reacting to them. Disruptions in critical parts of the intestine’s barrier cause permeability issues [6]. These barrier defects exacerbate the response of the underlying immune system, subsequently resulting in tissue damage [7]. These responses are regulated by multiple pro-inflammatory cytokines such as interferon gamma (IFN-γ), interleukin 1 beta (IL-1β), tumor necrosis factor alpha (TNF-α), and interleukin 6 (IL-6), all of which play a key role in disease progression [8,9]. Multiple pro-cytokines have critical roles in modulating intestinal inflammation and regulating intestinal permeability [8]. Previous studies prove that increased cytokines disrupt intestinal barrier function and cause barrier dysfunction in cultured epithelial monolayers [8,9,10].

Many natural products have curative value regarding IBD with minimal side effects [11]. One of these natural products, with various medical effects, is *Ziziphus jujuba* Miller (*Z. jujuba*). *Z. jujuba* is broadly distributed in Europe and Asia [12]. *Z. jujuba* fruit is rich in minerals, organic acids, volatile compounds, and fibers [13]. *Z. jujuba* also contains various phenolic acids [14] and flavonoids [15], which are responsible for its health benefits. In previous studies, *Z. jujuba* fruit showed various biological activities, including hepatoprotective, immunological [16], anticancer [17], antioxidant [18], and anti-inflammatory effects [19]. It is expected to have potential as a drug for treating IBD.

The aim of this study was to evaluate whether Ziziphus jujuba extract (ZJB) preserves the integrity of the intestinal epithelial barrier. Given the crucial role of the intestinal barrier in preventing IBD, understanding the protective effects of ZJB could provide valuable insights into potential therapeutic strategies. This study’s objective was to investigate the protective effects of ZJB on the intestinal barrier and elucidate its mechanism of action, particularly focusing on tight junction activation.

## 2. Materials and Methods

### 2.1. Extracts of Sample

*Z. jujuba* was purchased at Herbal Wholesale Market (Yeongcheon, Republic of Korea), and 30 g of dried *Z. jujuba* were ground to a powder, added to 70% ethanol (300 mL), and then extracted by shaking in an incubator at 100 rpm and 40 °C for 24 h. Following filtration using a 150 μm testing sieve (Retsch, Haan, Germany), the extracts were evaporated, lyophilized to concentrate them, and then kept at −20 °C. For the experiments, the freeze-dried *Z. jujuba* extract (ZJB) powder (10 mg) was dissolved in 1 mL of deionized distilled water (DW, *v*/*v*) and filtered using a 0.22 μm disk filter.

### 2.2. Preparation of Standard Solutions and Samples

A standard stock solution was prepared using DW; 1 mg/mL ZJB samples were properly weighted and diluted in DW. For all the standards, ZJB was stored at 4 or −20 °C. All the working solutions were filtered using a 0.45 μm syringe membrane filter (GVS filter technology, Sanford, ME, USA) before injection for high-performance liquid chromatography (HPLC) analysis.

### 2.3. Cell Culture and Treatments

Caco2 cells (KCLB, Seoul, Republic of Korea) were cultured at 37 °C in a humidified chamber under 5% CO_2_ in minimum essential medium (MEM) containing 10% fetal bovine serum (FBS) and 1% penicillin. Then, the cells were cultured using 0.25% trypsin at approximately 80–90% confluence. In our experiments, Caco2 cells were incubated with IL-6 (50 ng/mL) in the presence or absence of various concentrations of ZJB and incubated for 24 h.

### 2.4. Cell Viability Assay

Cell viability was evaluated through a Cell Counting Kit-8 (CCK-8; BIOMAX, Gyeonggi, Republic of Korea). A total of 1 × 10^4^ Caco2 cells were seeded into a 96-well plate. The CCK-8 assay was used to evaluate the cell viability following ZJB treatment. Caco2 cells were incubated for 24 h at concentrations of 10 to 300 μg/mL of ZJB. After that, 10 μL of the CCK-8 solution was added to each well, which was incubated for 1 h in an incubator. The absorbance of the CCK-8 solution was read at 450 nm; each sample was analyzed using the Synergy H1 multi-mode microplate reader (BioTek, Winooski, VT, USA).

### 2.5. DPPH (2,2-Diphenyl-1-picrylhydrazyl) Radical Cation Assay

The DPPH assay was used to assess the free radical scavenging capacity of the ZJB. DPPH is utilized as a reagent, which provides a straightforward and accurate method for titrating the oxidizable groups of natural or synthetic antioxidants. An amount of 190 μL of 0.2 mM DPPH methanolic solution was loaded into each well of a 96-well plate, followed by 10 μL of the sample, ascorbic acid (AA), or solvent for the blanks. The mixture was reacted at 37 °C for 30 min, and the absorbance at a wavelength of 517 nm was measured using the Synergy H1 multi-mode microplate reader (BioTek, Winooski, VT, USA). The radical remaining was calculated by the equation to percentage:

DPPH radical remaining (%) = 100 − 100 (Sample A/Blank A)

where Blank A is the absorbance of the blank and Sample A is the absorbance of the sample at 517 nm.

### 2.6. ABTS (2,2′-Azinobis-(3-ethylbenzothiazoline-6-sulfonate) Radical Cation Assay

The ABTS was produced by an oxidation procedure via potassium persulfate. The radical stock solution was prepared immediately before use to ensure freshness. The blue-green ABTS radical solution’s concentration was adjusted with methanol to get an absorbance of 0.70 ± 0.02 at 734 nm. To 190 μL of this ABTS solution, 10 μL of the sample, AA, or solvent was added into a 96-well plate. The mixture was reacted for 5 min at 37 °C, and it was measured at 734 nm using the Synergy H1 multi-mode microplate reader (BioTek, Winooski, VT, USA). The radical remaining was calculated by the equation to percentage:

ABTS radical remaining (%) = 100 − 100(Sample A/Blank A)

where Blank A is the absorbance of the blank and Sample A is the absorbance of the sample at 734 nm.

### 2.7. RNA Extraction and Quantitative PCR

Cell RNA was extracted by an RNA extraction kit (Qiagen, Hilden, Germany) according to the manufacturer’s instructions. The mRNA expression levels of TNF-α, C-C chemokine ligand 5 (CCL5), C-C chemokine ligand 17 (CCL17), and glyceraldehyde-3-phosphate dehydrogenase (GAPDH) in confluent Caco2 cell monolayers were assessed using the Biorad real-time PCR (qPCR) system (Biorad, Hercules, CA, USA) and Go Taq^®^ qPCR Master Mix. The primer sequences (NKMAX, Seongnam, Geonggi, Republic of Korea) are shown in Table 1.

### 2.8. Measurement of Trans-Epithelial Electrical Resistance (TEER) and Epithelial Permeability Assay

For the TEER and fluorescein isothiocyanate-dextran 4kDa (FITC-Dextran 4; Merck, Darmstadt, Germany) permeability assays, Caco2 cells were seeded, at 1 × 10^5^ cells, on insert well filter chambers in 24-well plates (0.4 μm pore size; SPL, Gyeonggi, Republic of Korea); these were cultured for 21 days, and the medium was replaced every day. The TEER value was determined every 6 h for 24 h using an EVOM3 Epithelial Volt/Ohm Meter (World Precision Instrument, Sarasota, FL, USA). For the FITC-Dextran 4 permeability assay, cells were washed with PBS (pH 7.4). Then, we added 1 mg/mL of FITC-Dextran 4 in PBS on the apical side and 1 mL of PBS on the basolateral side; these were then incubated at 37 °C for 1 h. Amounts of 100 μL of PBS were collected from the basolateral side, and the fluorescent absorbance was measured with a wavelength of excitation of 490 nm and emission at 520 nm.

### 2.9. Immunofluorescence (IF) Staining

The cells were cultured on slides with 4-well chambers (SPL, Gyeonggi, Republic of Korea) for 3 weeks; after the treatment, IF staining was used to validate the expression of Zonula occludens (ZO)-1 and occludin. The slide was washed with PBS and fixed with 4% paraformaldehyde in PBS for 10 min at 24 °C. The cells were permeabilized with 0.25% triton-X100 in PBS for 10 min. Then, they were incubated with blocking buffer containing 1% bovine serum albumin (BSA) in 0.1% Tween-20 in PBS for 30 min. Then, 200 μL of the anti-ZO-1 rabbit and anti-occludin mouse polyclonal antibodies (1:100, Invitrogen, Waltham, MA, USA) were added and incubated at 4 °C overnight. The cells were rinsed with PBS and incubated for 1 h by blocking the light with 200 μL of goat anti-mouse IgG and goat anti-rabbit IgG secondary antibodies, which were conjugated to FSD^TM^ 488 (1:100, BioActs, Incheon, Republic of Korea) and FSD^TM^ 594 (1:100, BioActs, Incheon, Republic of Korea). Finally, the slide wells were washed with PBS to remove the remaining water; then, one drop of mounting solution was added, and slides were covered with glass (DWK Life Sciences, Beijing, China). After that, TJ proteins were visualized, and images were obtained using a cell imaging system (Cytation 7, BioTek, Winooski, VT, USA).

### 2.10. Protein Extraction and Western Blotting

Caco2 cells, at 8 × 10^5^ per well, were seeded in 60π cell culture plates. After treatment for 24 h, Caco2 monolayers were rinsed with PBS, and lysed with appropriate amounts of radio-immunoprecipitation assay (RIPA) lysis buffer (Thermo Scientific Fisher, Waltham, MA, USA) containing an Xpert protease inhibitor cocktail solution (100×, GenDEPOT, Katy, TX, USA). Cell lysates were centrifuged for 15 min at 13,000 rpm at 4 °C. The supernatants were transferred, and the proteins were quantified using the bicinchoninic acid (BCA) protein quantification kit (BIOMAX, Gyeonggi, Republic of Korea). SDS-PAGE sample loading buffer (5×; ELPis, Daejeon, Republic of Korea) was added to the lysate containing 20 µg of protein, and this was heated at 80 °C for 12 min, after which equal amounts of protein for each sample were separated on a 10% SDS-PAGE gel. The proteins were transferred from the SDS-PAGE gel to a polyvinylidene fluoride (PVDF) membrane (GVS, USA). Next, the membranes were blocked in 5% skimmed milk in TBS containing 0.1% Tween-20 (TBS-Tween; washing buffer; WB) for 2 h, and then incubated with appropriate primary antibodies (inducible nitric oxide synthase (iNOS) at 1:1000, cyclooxygenase-2 (COX-2) at 1:1000, anti-ZO-1 at 1:1000, anti-occludin at 1:2000, anti-β-actin at 1:1000) overnight at 4 °C on a rocker with slow shaking. After 24 h, and after being washed with WB, the membranes were incubated with appropriate secondary antibodies for 2 h at 24 °C. Protein bands were detected by the SuperSignal™ West Pico PLUS Chemiluminescent Substrate Kit (Thermo Scientific Fisher, Waltham, MA, USA) and analyzed using an imaging system (Shenhua Science Technology, Hangzhou, China). The bands were normalized by β-actin.

### 2.11. Experimental Animals

C57BL/6J male mice, 6 weeks old (25–30 g), were purchased from Samtako Inc. (Osan, Republic of Korea). The mice were housed in cages under 22 ± 2 °C and 55 ± 5% relative humidity conditions with a 12 h/12 h light and dark cycle; the mice had free access to drinking water and a standard diet (Orientbio Inc., Sungnam, Republic of Korea). All the experimental animal procedures were approved by the Korea Institute of Oriental Medicine (KIOM, Daegu, Republic of Korea) Institutional Animal Care and Use Committee (KIOM-D-19-007) and were conducted in accordance with the guidelines of the National Institutes of Health (NIH publication).

### 2.12. Dextran Sodium Sulfate (DSS)-Induced Colitis and Treatment

**Figure 1:**
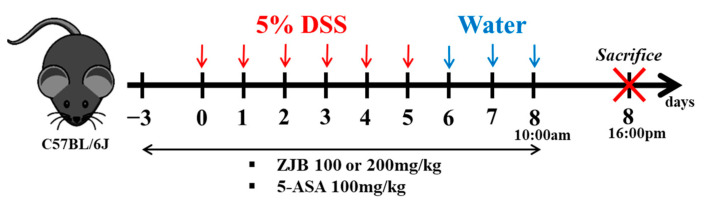
Animal experiment schedule.

All mice were randomly divided into five groups (*n* = 8 per group): The vehicle group, the DSS group, the ZJB 100 mg/kg + DSS group (ZJB 100 mg/kg group), the ZJB 200 mg/kg + DSS group (ZJB 200 mg/kg group), and the 5-aminosalicylic acid (5-ASA, as positive control) 100 mg/kg + DSS group (5-ASA group). Colitis induction involved administering 5% DSS (wt/vol) (MP Biomedicals, colitis grade, molecular weight of 36–50 kDa) to all mice, except for the vehicle group, from day 0 to day 5 using drinking bottles. Subsequently, the DSS solution was replaced with DSS-free water from day 6 to day 8 using the same method. The mice in the vehicle group were provided with normal water throughout. The mice in the ZJB and 5-ASA groups were orally administered 100 or 200 mg/mL of ZJB or 100 mg/mL of 5-ASA for 12 days (Figure 1). Before oral gavage with ZJB and 5-ASA, the body weights were measured daily. On the last day of experiments, serum was collected through respiratory anesthesia (isoflurane, USA), and the mice were sacrificed by cervical dislocation to obtain colon tissue.

### 2.13. Disease Activity Index (DAI) and Scoring

DAI scoring was measured daily after the administration of 5% DSS (from day 0); this was based on body weight loss, stool firmness, and hemorrhaging. Scores in each category, ranging from 0 (healthy) to 4 (most severe), were tallied as follows: Weight loss: 0 (no loss), 1 (1–5%), 2 (5–10%), 3 (10–20%), and 4 (>20%); stool firmness: 0 (normal), 1–2 (loose stool), 3 (diarrhea), and 4 (no stools); rectal hemorrhaging: 0 (none), 1 (slightly bloody and slightly light and dark brown), 2 (modest bloody and dark brown), 3 (more hemorrhaging and diarrhea red), and 4 (gross hemorrhaging and bright red, blood in the whole colon). With the formula, DAI = body mass index score + stool shape score + bleeding score, the value for the combined score of the three results was assigned as the DAI value.

### 2.14. Endoscopy and Hematoxylin and Eosin (H&E) Staining

To observe the histological changes in the intestinal epithelial barrier in DSS-induced mice, images were acquired using an Olympus mini-endoscope (length: 670 mm, diameter: 2.8 mm; Tokyo, Japan) on mice under anesthesia. After the endoscopy procedure, the obtained tissues were fixed with 4% paraformaldehyde solution and embedded in a paraffin block. After that, tissues were sectioned using a microtome. Histological sections were stained using H&E.

### 2.15. Cytokine Detection Using Enzyme-Linked Immunosorbent (ELISA) Assay

The cell medium was obtained after 24 h to measure the cytokine concentration. The obtained serum samples were centrifuged at 12,000 rpm and 20 °C for 15 min. The concentrations of interleukin 2 (IL-2), IL-6, and TNF-α were evaluated using the ELISA kit according to the manufacturer’s recommendations (R&D Systems, Minneapolis, MN, USA). The absorbance was measured using the Synergy H1 microplate spectrophotometer (BioTek, Winooski, VT, USA) at 490 nm. The cytokine levels were extrapolated from the IL-6 and TNF-α standard curves from 0 to 2000 pg/mL. All results were expressed as pg/mL.

### 2.16. Liquid Chromatography with Tandem Mass Spectrometry (LC-MS/MS) Analysis

An LC-MS/MS analysis of ZJB was performed using a Nexera ultra-performance liquid chromatography (UPLC) system (Shmadzu, Kyoto, Japan) coupled with an ultra-quadrupole time of flight LC-MS/MS system (X500R QTOF MS system, AB SCIEX, Framingham, MA, USA) with a Pronto SIL 120-5-C18 SH (150 × 4.6 mm, 5 μm) column (Bischoff chromatography, Leonberg, Germany). A gradient of water and 0.1% formic acid in acetonitrile (ACN) was used for each mobile phase; for electrospray ionization-mass spectrometry (ESI), 5% to 90% water and 10% to 95% ACN were applied for 70 min at 35 °C and flowed 0.5 mL/min. The samples were measured in positive ion mode. Data were acquired using SCIEX OS software 3.0 (SCIEX, Framingham, MA, USA).

### 2.17. Statistical Analysis

All the statistical analyses were performed with GraphPad Prism 8.0 (GraphPad Software, San Diego, CA, USA). One-way or two-way factorial analysis of variance (ANOVA) was used to analyze the difference. The results are presented as the mean ± standard error of the mean (SEM), and a *p* value < 0.05 was considered statistically significant.

## 3. Results

### 3.1. ZJB Improved the Disruption of the Intestinal Epithelial Barrier by IL-6

#### 3.1.1. Cytotoxic Effects of IL-6 and ZJB in Caco2 Cells

**Figure 2:**
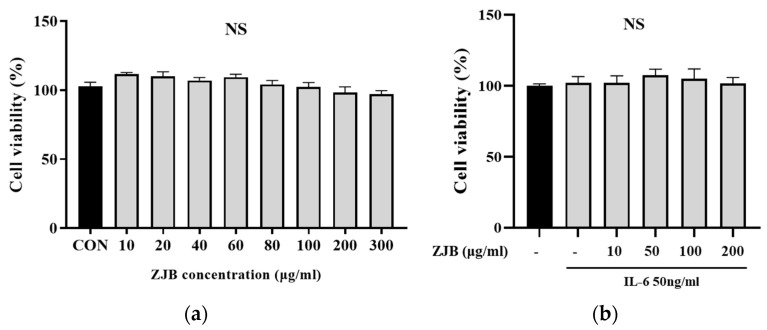
Cell viability of ZJB in Caco2 cells. The cell viability was determined using the CCK-8 kit. (**a**) ZJB was used at various concentrations (0, 10, 20, 40, 60, 80, 100, 200, and 300 μg/mL) for 24 h. (**b**) ZJB at 10, 50, 100, and 200 μg/mL, with pretreatment for 1 h with 50 ng/mL of IL-6 added and incubation for 24 h (*n* = 10). All the data are representative of at least three independent experiments. Statistical analysis was performed according to one-way ANOVA with Dunnett’s multiple comparison test. Data indicate the mean ± SEM. Not significant (NS) versus control group. CON: control.

To determine the effects of ZJB in Caco2 cells, cell viability was evaluated at various ZJB concentrations using the CCK-8 assay after incubating the cells for 24 h in the presence or absence of IL-6 (50 ng/mL). The results showed that ZJB concentrations ranging from 10 to 300 µg/mL with or without IL-6 were not cytotoxic to Caco2 cells (Figure 2). Therefore, ZJB was considered safe and was used for further experiments.

#### 3.1.2. ZJB Has an Antioxidant Effect

**Figure 3:**
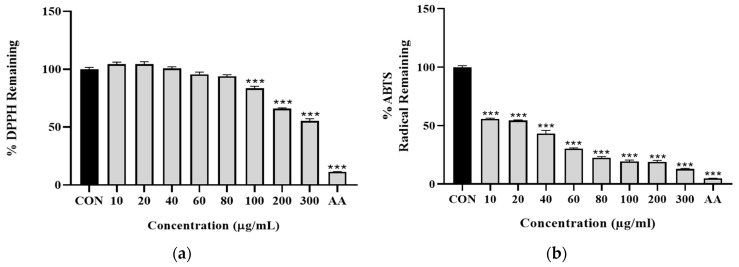
Effects of ZJB in regulating inflammation. (**a**) Effects of antioxidants of ZJB according to DPPH assay. The DPPH free radical scavenging activity of the test sample was measured with a 0.091 mM DPPH methanolic solution. (**b**) Effects of antioxidants of ZJB according to ABTS assay. The ABTS radical scavenging activity of the ZJB was measured with an O.D 0.7 nm ABTS solution (*n* = 10). All data are representative of at least three independent experiments. Statistical analysis was performed according to one-way ANOVA with Dunnett’s multiple comparison test. Data indicate the mean ± SEM. *** *p* < 0.001 versus control group. CON: control, AA: ascorbic acid (100 μM).

Measuring the antioxidant efficacy of ZJB through the DPPH assay showed a significant antioxidant effect for a concentration of 100 µg/mL (Figure 3a). Additionally, measuring the antioxidant efficacy via an ABTS assay showed that the remaining radical was significantly decreased from 10 μg/mL (Figure 3b). Based on these results, we determined that ZJB has excellent antioxidant effects.

#### 3.1.3. Inflammatory Cytokines and Chemokines Are Regulated by ZJB

**Figure 4:**
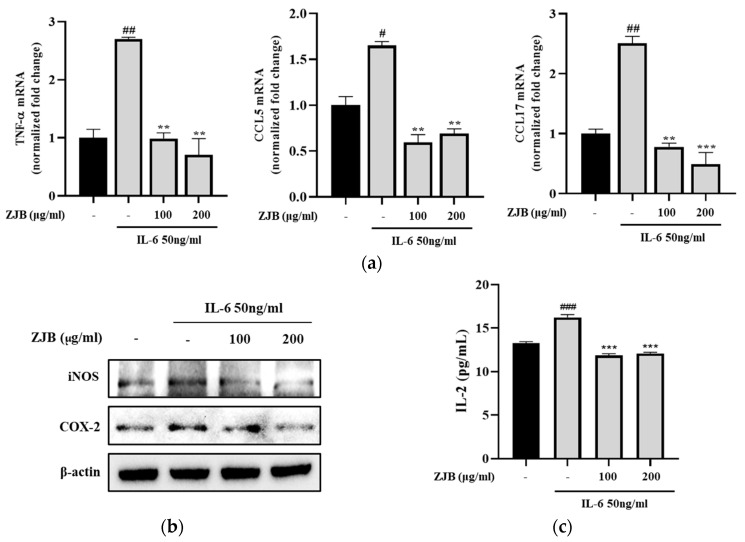
ZJB has an anti-inflammatory effect on IL-6-induced Caco2 cells. Caco2 cells were treated with 50 ng/mL IL-6 in the presence of 100 or 200 μg/mL ZJB for 24 h. (**a**) Effect of ZJB on inflammatory cytokine/chemokine mRNA expression levels in IL-6-induced Caco2 cells. The relative mRNA expressions of TNF-α, CCL5, and CCL17 were detected via qPCR analysis. (**b**) ZJB inhibited IL-6-induced activation of inflammation markers. The expression levels of the iNOS and COX-2 proteins were detected via Western blotting. (**c**) ZJB inhibited inflammatory cytokine secretion in Caco2 cells. The concentration of IL-2 was measured using an ELISA kit. All data are representative of at least three independent experiments. Statistical analysis was performed according to one-way ANOVA with Dunnett’s multiple comparison test. Data indicate the mean ± SEM (*n* = 5). # *p* < 0.05, ## *p* < 0.01, and ### *p* < 0.001 versus control group; ** *p* < 0.01 and *** *p* < 0.001 versus IL-6-treated group.

To evaluate the expression of genes for inflammatory cytokines/chemokines following ZJB treatment, qPCR was used to measure the levels of TNF-α, CCL5, and CCL17. The mRNA levels of TNF-α, CCL5, and CCL17 were significantly increased in the IL-6 group compared with the control. However, their expression levels were down-regulated by treatment with 100 or 200 μg/mL ZJB (Figure 4a). Compared with the IL-6 group, the inflammation protein markers iNOS and COX-2 were reduced by ZJB in a concentration-dependent manner (Figure 4b). Additionally, the secretion of inflammatory cytokines by Caco2 cells was measured during ZJB treatment, and the results showed that the concentration (pg/mL) of IL-2 was reduced in the 100 and 200 μg/mL ZJB groups (Figure 4c). These results indicated that ZJB has a strong anti-inflammatory effect that can suppress inflammatory responses by down-regulating IL-6-induced inflammation in Caco2 cells.

#### 3.1.4. ZJB Maintains the Function of Intestinal Epithelial Cell Monolayers

**Figure 5:**
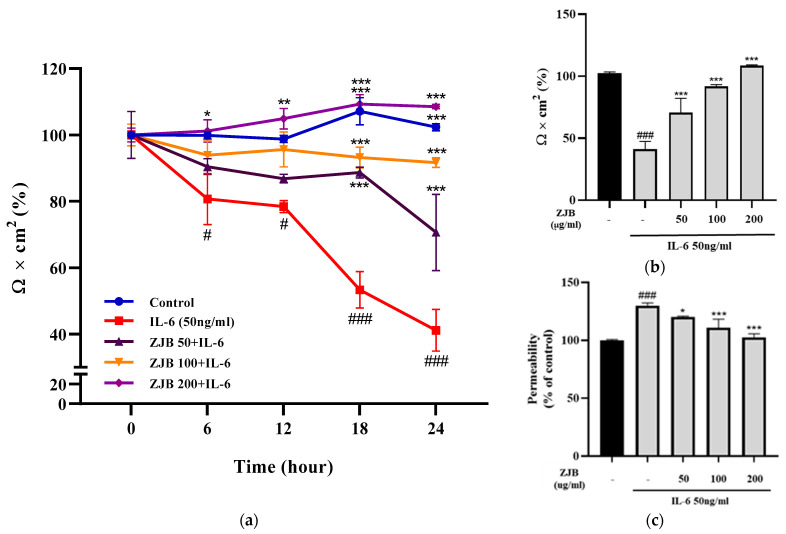
Effect of regulation of epithelial barrier function by ZJB. (**a**) TEER values for ZJB-treated Caco2 monolayers. (**b**) Percentage levels of TEER values at 24 h. (**c**) Permeability to FITC-Dextran 4 of ZJB-treated Caco2 monolayers. All data are representative of at least three independent experiments. Statistical analysis was performed according to one-way ANOVA with Dunnett’s multiple comparison test. Data indicate the mean ± SEM (*n* = 5). # *p* < 0.05 and ### *p* < 0.001 versus control group; * *p* < 0.05, ** *p* < 0.01, and *** *p* < 0.001 versus IL-6-treated group.

To evaluate how well ZJB protects epithelial barrier function, the latter was measured using TEER and fluxes of FITC-Dextran 4 permeability. The TEER values were measured every 6 h. In the IL-6 group, the TEER values tended to gradually decrease over 24 h and decreased sharply at 18 h (53.31%) compared to 12 h (78.44%). After 24 h, these values decreased to 41.14% compared to the control (100%). However, ZJB protected against the decrease in TEER value induced by IL-6. The decrease in TEER value induced by IL-6 was inhibited in a manner dependent on the ZJB concentration. In the 100 μg/mL ZJB group, the value was 91.69% at 24 h, lower than the control (102.33%). In the case of 200 μg/mL ZJB, it showed a tendency to increase over time, and the value was similar to the control (102.33%) at 24 h (108.53%) (Figure 5a,b). Permeability also showed the same tendency (Figure 5c). The results show that ZJB protected against the impairment of barrier dysfunction induced by IL-6.

#### 3.1.5. ZJB Prevents Morphological Disruption of TJ Proteins Induced by IL-6

**Figure 6:**
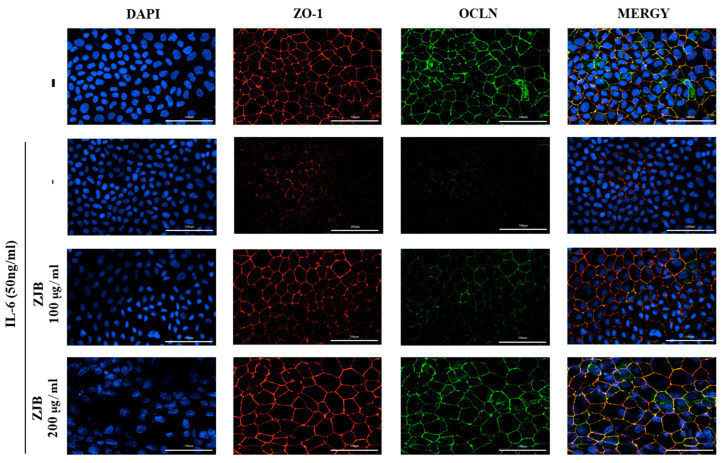
Effects of preventing TJ protein interruption with ZJB. Representative immunostaining for ZO-1 (red) and occludin (green) protein in Caco2 monolayer (*n* = 5). All data are representative of at least three independent experiments. Scale bar = 100 μm.

The increased secretion of pro-inflammatory cytokines/chemokines might cause paracellular permeability due, primarily, to the disruption of TJ proteins such as ZO-1 and occludin. To verify the integrity of intercellular junctional complexes, we performed an IF assay. Normal circumstances resulted in ZO-1 and occludin being localized to the cell membrane, where they appeared without interruption surrounding the cell boundaries. However, IL-6 caused the expression of ZO-1 and occludin at the cell border to be discontinuous and interrupted. We observed that the expression of ZO-1 and occludin became clearer without interruption in a concentration-dependent manner with ZJB treatment. In particular, 200 μg/mL ZJB clearly showed TJ protein expression at cell boundaries (Figure 6).

#### 3.1.6. ZJB Prevents the Reduction in TJ Proteins Induced by IL-6

**Figure 7:**
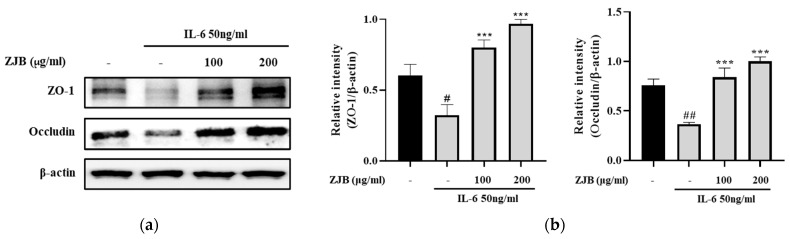
Effects of protection of TJ proteins by ZJB. (**a**) The expression of TJ proteins. (**b**) Expression levels of ZO-1 and occludin were normalized to β-actin. All data are representative of at least three independent experiments. Statistical analysis was performed according to one-way ANOVA with Dunnett’s multiple comparison test. Data indicate the mean ± SEM (*n* = 5). # *p* < 0.05, ## *p* <0.01 versus control group; *** *p* < 0.001 versus IL-6 group.

To confirm the effect of ZJB on TJs, which play an important role in maintaining the tight intestinal barrier, the amount of change in TJ protein was analyzed through Western blot analysis. As a result, ZO-1 and occludin expression was reduced in the IL-6 group compared with the control. However, pretreatment with ZJB significantly increased ZO-1 and occludin expression in a concentration-dependent manner (Figure 7). This means that ZJB inhibited the down-regulation of TJs in IL-6-induced Caco2 cells.

### 3.2. ZJB Protected against Impairment of the Intestinal Barrier and TJs in DSS-Induced Mice

#### 3.2.1. ZJB Restores Weight Loss and Colon Shortening Caused by DSS

**Figure 8:**
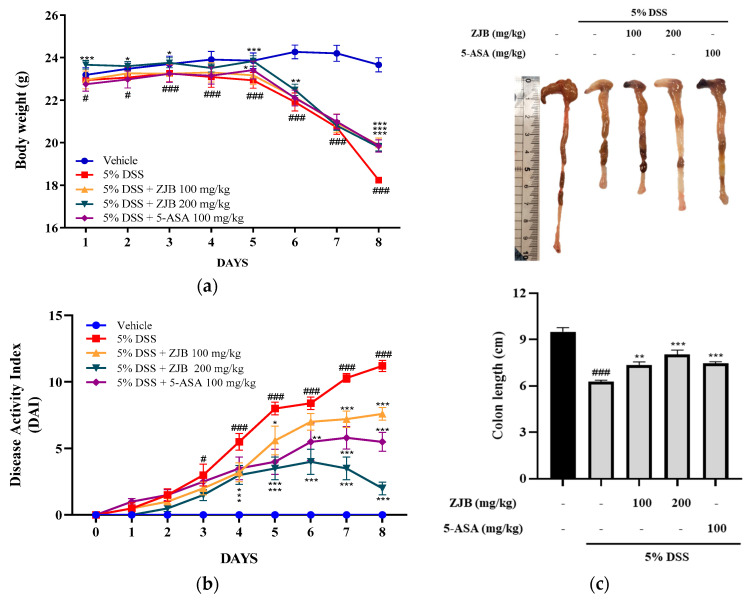
The symptoms of DSS-induced colitis mice can be ameliorated by ZJB administration. (**a**) Changes in body weight (%). (**b**) Disease activity index (DAI) scores following ZJB or 5-ASA administration during experimental period in DSS-induced mice. (**c**) Colon length shortening during DSS-induced periods. Statistical analysis was performed according to one-way or two-way ANOVA with Dunnett’s multiple comparison test. Data indicate the mean ± SEM (*n* = 8). # *p* < 0.05 and ### *p* < 0.001 versus vehicle group; * *p* < 0.05, ** *p* < 0.01, and *** *p* < 0.001 versus DSS-induced group.

The main signal manifestations of UC in DSS are directly toxic to the colonic epithelium, leading to serious illness characterized by a shortening of the colon and sustained weight loss [20]. Upon investigating the therapeutic effect of oral ZJB administration on acute colitis induced by DSS, a decrease in body weight and an increase in DAI were confirmed in DSS-induced mice. These clinical symptoms improved with the administration of ZJB compared to the vehicle group (Figure 8a,b). Furthermore, consistent with the body weight results, colon lengths were decreased in the DSS-induced group compared with the vehicle group. ZJB suppressed colon length loss (Figure 8c).

#### 3.2.2. ZJB Reduces Cellular Infiltration and Improves Damaged Colon Tissues

**Figure 9:**
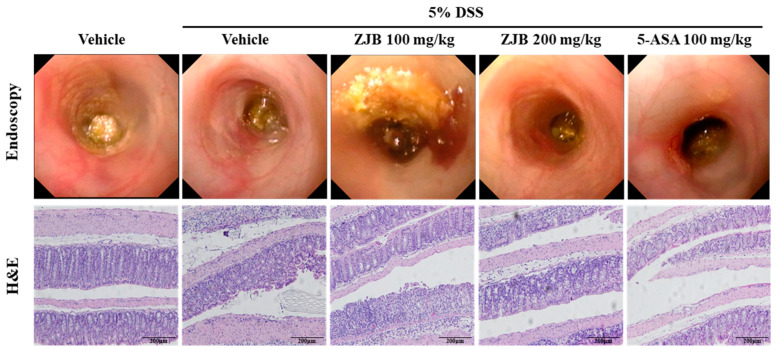
ZJB protected against damage to colon epithelial intestinal tissue in the DSS-induced colitis model. Confirmation of DSS-induced mucosal damage using a mini-endoscope (*n* = 8). Representative H&E-stained colon section images are shown at 200× magnification (*n* = 4).

To evaluate the effect of ZJB in DSS-induced mice, the morphological and histological changes under colitis in the large intestine were observed through endoscopy and H&E staining. Colonic tissues from the vehicle group exhibited a normal structure. The colonic tissues of the DSS group showed serious morphological and histological changes, including massive epithelial destruction, crypt abscesses, and goblet cell loss. However, this histopathological damage induced by DSS was significantly inhibited by the administration of 200 µg/mL of ZJB (Figure 9). These results suggest that ZJB may improve DSS-induced colitis.

#### 3.2.3. ZJB Regulates the Expression of TJ Protein in DSS-Induced Mice

**Figure 10:**
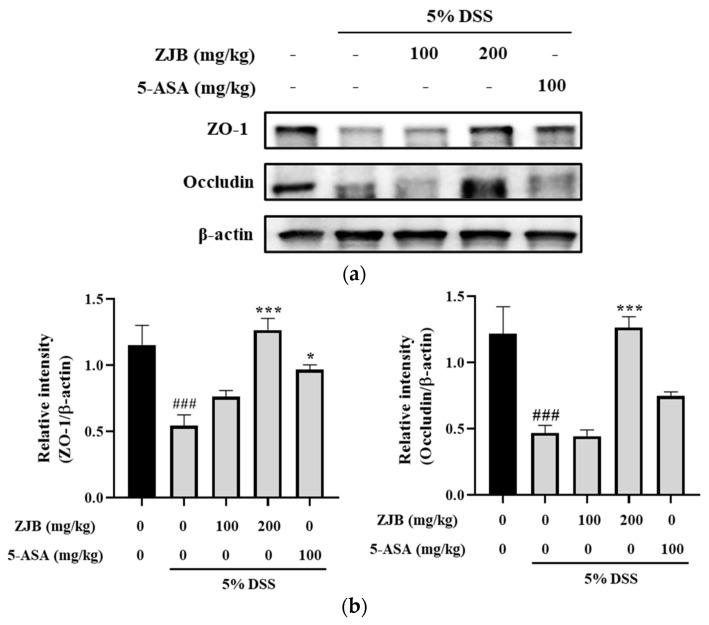
ZJB improved intestinal TJs in the DSS-induced colitis model. (**a**) The protein levels of ZO-1 and occludin in colonic tissues were detected via Western blotting. (**b**) Normalization of ZO-1 and occludin expression to β-actin. Western blot analysis was conducted on colon tissue (*n* = 4). Statistical analysis was performed according to one-way ANOVA with Dunnett’s multiple comparison test. Data indicate the mean ± SEM. ### *p* < 0.001 versus vehicle group; * *p* < 0.05 and *** *p* < 0.001 versus DSS-induced group.

To evaluate the effect of ZJB on intestinal TJ dysfunction in mice, Western blot analysis was performed. As a result, the ZO-1 expression level was reduced in the DSS-induced group; this was increased with ZJB treatment. In addition, the protein expression levels of ZO-1 and occludin decreased in DSS-induced colitis. However, this was recovered by ZJB treatment. These results indicate that ZJB prevented the down-regulation of TJ proteins in DSS-induced colitis (Figure 10).

#### 3.2.4. ZJB Inhibits Pro-Inflammatory Cytokine Secretion in DSS-Induced Colitis Mice

**Figure 11:**
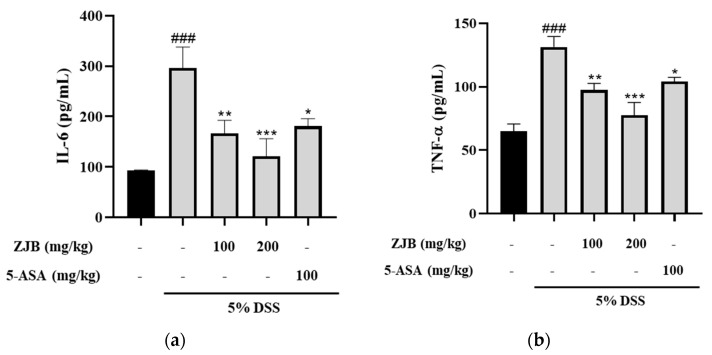
Effects of ZJB on pro-inflammatory cytokine levels in serum in DSS-induced colitis model. (**a**) The serum levels of IL-6 and (**b**) TNF-α were detected using the ELISA kit. Statistical analysis was performed according to one-way ANOVA with Dunnett’s multiple comparison test. Data indicate the mean ± SEM (*n* = 8). ### *p* < 0.001 versus vehicle group; * *p* < 0.05, ** *p* < 0.01, and *** *p* < 0.001 versus DSS-induced group.

Increased inflammatory mediators are an additional cause of intestinal tissue damage and decreased mucosal barrier function. To evaluate the effects of ZJB on inflammation and TJs, we determined the levels of the inflammatory mediators TNF-α and IL-6, showing that both were increased in the DSS group compared with the vehicle group. However, ZJB administration reduced the relative levels of IL-6 and TNF-α (Figure 11).

### 3.3. Analysis of LC–MS/MS to Identify Active Compounds in ZJB

**Figure 12:**
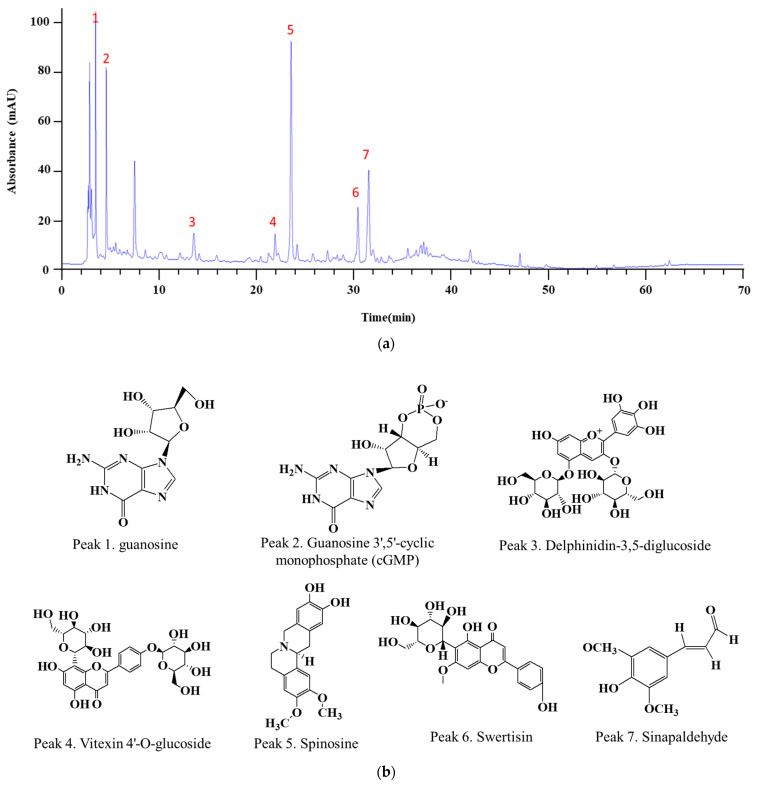

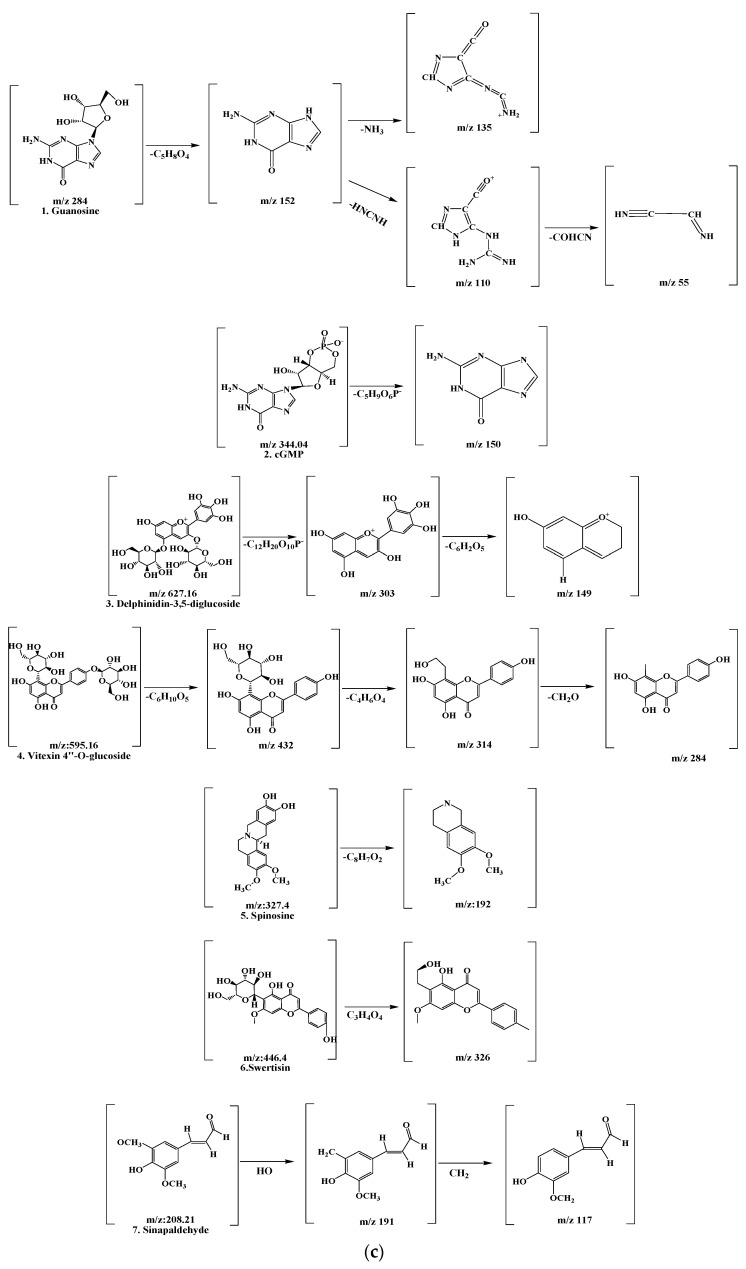
The LC-MS/MS chromatogram and the chemical structures of seven markers of ZJB (**a**). Chromatogram of ZJB analysis through LC-MS/MS: This analysis was separated by gradient elution. The sample injection volume was 10 μL, and the flow rate was 1.0 mL/min. (**b**) The chemical structures of seven markers of ZJB. (**c**) Proposed fragmentation patterns for guanosine (*m*/*z* 284), cGMP (*m*/*z* 344.04), delphinidin-3,5-diglucoside (*m*/*z* 627.16), vitexin 4″-O-glucoside (*m*/*z* 595.16), spinosine (*m*/*z* 327.4), swertisin (*m*/*z* 446.4), and sinapaldehyde (*m*/*z* 208.21).

The constituents of ZJB extracts were analyzed through LC-MS/MS analysis: Guanosine (1) [21], guanosine 3′,5′-cyclic monophosphate (cGMP) (2) [22], delphinidin-3,5-diglucoside (3) [23], vitexin 4′-O-glucoside (4) [24,25], spinosine (5) [26], swertisin (6) [27], and sinapaldehyde (7) [21], respectively, based on the retention times and mass spectra (Figure 12 and Table 2).

## 4. Discussion

Intestinal barrier function defects are associated with GI tract diseases [28]. Therefore, the intestinal barrier’s function plays a central role in the onset and progression of UC [29]. The intestinal epithelial barrier serves as the initial layer of defense against various kinds of bacterial infection and is responsible for maintaining intestinal mucosal homeostasis [30]. The dysfunction of the intestinal epithelial barrier provides growing evidence that increases in intestinal permeability play a pathogenic role in diseases such as IBD [7,28].

IL-6 is a pleiotropic cytokine that is involved in antigen-specific immune responses and has pro-inflammatory properties [31,32]. The excessive production and dysregulation of IL-6 and its signaling pathway have been known to contribute significantly to IBD etiology. In vitro studies suggest that cytokines, such as IL-6, IL-1β, and TNF-α, induced inflammation and caused intestinal barrier dysfunction in epithelial cells [31,33]. This means that IL-6 signaling pathways play an essential role in IBD pathogenesis. Therefore, pro-inflammatory cytokines, such as TNF-α, IL-6, and IL-1β, and TJ proteins such as ZO-1 and occludin, are important targets in IBD treatment and in regulating intestinal barrier function [7,34]. Up until now, several medicines have been used in the treatment of UC, including 5-aminosalicylic acid (5-ASA), azathioprine (AZA), 6-mercaptopurine (6-MP), and cyclosporine [19]. However, UC medicines have side effects that cannot be ignored [19]. Various therapeutic strategies for ameliorating this condition are currently being studied. Among them, many researchers have reported on herbal medicines with validated therapeutic potential for protecting intestinal barrier function [35,36].

In this study, we established a colitis model, which is a well-known animal model of intestinal inflammation, using DSS, with a phenotype resembling human disease [37,38,39,40]. The effect of ZJB on intestinal barrier dysfunction recovery was evaluated, focusing on the regulation of the secretion of pro-inflammatory mediators and modulation of TJ proteins in IBD treatment. The cytotoxicity depends on the type or concentration of herbal medicines, so it is important to study toxic side effects. We confirmed that ZJB is non-cytotoxic from low doses to high doses in Caco2 cells (Figure 2a) and that, over 100 µg/mL, ZJB has an antioxidant effect (Figure 3a,b). Although no side effects were observed with the tested doses of ZJB, we noted the absence of a dose–response effect on pro-inflammatory cytokine expression. Nonetheless, ZJB treatment exerted significant dose-dependent effects on other key parameters, including dextran permeability, TEER values, and protein expression levels, in both cell and animal models, highlighting its potential therapeutic efficacy.

It is known that, in the onset and progression mechanisms of IBD, the excessive production of inflammatory cytokines and chemokines and the activation of immune cells play pivotal roles [29,41,42,43]. iNOS plays a crucial role in the onset and progression of active UC [44], inducing the excessive production of nitric oxide (NO), which exacerbates inflammation [45]. CCL5 and CCL17 are known to be increased under inflammatory responses; it is also known that an increase in inflammatory chemokines promotes IL-2 expression [46,47]. IL-2 is a critical pro-inflammatory factor because it promotes the expansion of T cells, the enhancement of natural killer (NK) cells, and the strengthening of the anti-tumor immune response [48]. In this study, we observed an up-regulation of inflammatory mediators, including IL-2; TNF-α, CCL5, and CCL17 mRNA expression; and iNOS and COX-2 protein expression in IL-6-treated Caco2 cells. However, ZJB significantly inhibited the expression of these factors (Figure 4).

In IBD patients, the levels of IL-6 and TNF-α production are significantly greater in the serum and tissues, and enhanced local or systemic inflammation disrupts TJs and leads to worsened colitis [49]. Pro-inflammatory cytokines increase paracellular permeability, impairing the epithelial barrier [45,50]. In this study, barrier disruption by IL-6 resulted in increased FITC-Dextran 4 permeability and decreased TEER values (Figure 5). ZJB improved the impaired TEER values and permeability (Figure 5). These findings indicate that ZJB has an inhibitory effect on inflammatory cytokine production and that it can allow the preservation and restoration of TJ integrity. TJs are essential for maintaining barrier function, with ZO-1 and occludin recognized as primary proteins [51]. We observed a reduction in the expression of ZO-1 and occludin due to IL-6- and DSS-induced damage to the colonic epithelium. However, ZJB restored TJ protein disruption (Figure 6, Figure 7, and Figure 10). As mentioned before, colitis is a pathological condition characterized by inflammatory responses in the intestine and intestinal barrier function impairment. We determined that ZJB treatment restored UC clinical symptoms and intestinal shortening (Figure 8), as well as pathological injuries in the mucosal barrier (Figure 9). This relieving of symptoms is related to the up-regulation of TJ proteins and the down-regulation of inflammatory cytokines (Figure 10 and Figure 11).

*Z. jujuba* contains large amounts of total phenolic compounds and flavonoids [52]. Polyphenols exhibit pharmacological activities such as anti-inflammatory, antioxidant, and anti-tumor effects. Among the seven substances detected by LC-MS/MS analysis (Figure 5), delphinidin-3,5-diglucoside, vitexin 4′-O-glucoside, spinosine, and swertisin are flavonoids, and sinapaldehyde is a phenolic acid [17]. Additionally, two more active ingredients, guanosine and cGMP, play crucial roles in cell responses. A previous study reported that guanosine and cyclic guanosine monophosphate (cGMP) levels decreased in DSS-induced mice [53,54]. cGMP found in ZJB could contribute to preserving the integrity of the intestinal barrier by modulating TJ proteins; its signaling pathways have also been linked to the regulation of tight junction integrity, which protects the intestinal barrier [55]. In particular, cGMP is essential for maintaining mucosal homeostasis and suppressing inflammatory responses [55]. cGMP effector, as cGMP-dependent protein kinase C (PKG), is expressed in tissues throughout the intestine; it is known to affect both the epithelial and endothelial barriers [56]. PKGI and PKGII, PKG, isoforms are found in smooth muscle cells and the intestinal epithelium, respectively; they are responsible for regulating luminal fluid secretion and intestinal contractility, respectively [57]. GMP signaling inhibits intestinal inflammatory responses, partly through protecting epithelial barrier integrity [58,59]. Therefore, the presence of cGMP in ZJB could potentially enhance the integrity of the intestinal barrier by regulating tight junctions, thereby protecting against intestinal inflammation and dysfunction.

In summary, our study presents promising evidence regarding the efficacy of ZJB in treating IBD. Nonetheless, several limitations should be considered. One limitation of this study is that, while ZJB demonstrated a lack of cytotoxicity and impressive antioxidant effects, the specific mechanisms underlying its anti-inflammatory properties and its impact on intestinal barrier function require further elucidation. Additionally, while LC-MS/MS analysis identified several active ingredients in ZJB, additional studies are necessary to determine the contribution of each component to its therapeutic effects in IBD. Despite these limitations, our findings suggest the potential of ZJB as a natural and safe therapeutic agent for the management of IBD.

## 5. Conclusions

ZJB can effectively reduce IL-6- and DSS-induced colitis. Through this study, we proved ZJB’s ability to protect intestinal barrier function and ameliorate TJ impairment. Additionally, ZJB has been confirmed to be a safe, natural product; a high dose of ZJB better regulates the expressions of IL-6 and TJ proteins such as ZO-1 and occludin. These findings offer fresh perspectives on the mechanisms by which ZJB serves as a potential agent to ameliorate UC severity. Therefore, it can be incorporated as an effective food product and medical substance that effectively prevents UC and restores intestinal TJs.

## Figures and Tables

**Table 1:** Primer sequences.

Gene	Forward	Reverse
*TNF-α*	5′-ACATACTGACCCACGGCTTC-3′	5′-GCACTCACCTCTTCCCTCTG-3′
*CCL5*	5′-TGCTGCTTTGCCTACATTG-3′	5′-CACTTGGCGGTTCTTTCG-3′
*CCL17*	5′-CTGATGAGCCTCAGGTGACA-3′	5′-CCAGGATGCTCTCAGTCACA-3′
*GAPDH*	5′-GAAGGTGAAGGTCGGAGT-3′	5′-CATGGGTGGAATCATATTGGAA-3′

**Table 2:** Tentative identification of the chemical components of ZJB obtained from the UPLC-TOF-MS analysis.

Peak No.	Compound	Retention Time (min)	Formula	[M + H]^+^	MS/MS
1	Guanosine	3.51	C10H13N5O5	284	152, 135, 110, 55
2	Guanosine 3′,5′-cyclic monophosphate (cGMP)	4.59	C10H12N5O7P−	346	346, 152
3	Delphinidin-3,5-diglucoside	13.42	C27H31O17+	627	303, 149
4	Vitexin 4″-O-glucoside	21.96	C27H30O15	595	432, 314, 284
5	Spinosine	24.6	C19H21NO4	327	327, 192
6	Swertisin	31.3	C22H22O10	446	326
7	Sinapaldehyde	32.04	C11H12O4	209	191, 177

## Data Availability

The data presented in this study are available on request from the corresponding author.
